# Randomized DNA libraries construction tool: a new 3-bp ‘frequent cutter’ TthHB27I/sinefungin endonuclease with chemically-induced specificity

**DOI:** 10.1186/s12864-018-4748-0

**Published:** 2018-05-11

**Authors:** Daria Krefft, Aliaksei Papkov, Maciej Prusinowski, Agnieszka Zylicz-Stachula, Piotr M. Skowron

**Affiliations:** Department of Molecular Biotechnology, Faculty of Chemistry, University of Gdansk, Wita Stwosza 63, 80-308 Gdansk, Poland

**Keywords:** Restriction endonuclease-methyltransferase, Thermophile, Star activity, Specificity relaxation, Genomic libraries, DNA fragmentation

## Abstract

**Background:**

Acoustic or hydrodynamic shearing, sonication and enzymatic digestion are used to fragment DNA. However, these methods have several disadvantages, such as DNA damage, difficulties in fragmentation control, irreproducibility and under-representation of some DNA segments. The DNA fragmentation tool would be a gentle enzymatic method, offering cleavage frequency high enough to eliminate DNA fragments distribution bias and allow for easy control of partial digests. Only three such frequently cleaving natural restriction endonucleases (REases) were discovered: CviJI, SetI and FaiI. Therefore, we have previously developed two artificial enzymatic specificities, cleaving DNA approximately every ~ 3-bp: TspGWI/sinefungin (SIN) and TaqII/SIN.

**Results:**

In this paper we present the third developed specificity: TthHB27I/SIN(SAM) - a new genomic tool, based on Type IIS/IIC/IIG *Thermus*-family REases-methyltransferases (MTases). In the presence of dimethyl sulfoxide (DMSO) and S-adenosyl-L-methionine (SAM) or its analogue SIN, the 6-bp cognate TthHB27I recognition sequence 5’-CAARCA-3′ is converted into a combined 3.2–3.0-bp ‘site’ or its statistical equivalent, while a cleavage distance of 11/9 nt is retained. Protocols for various modes of limited DNA digestions were developed.

**Conclusions:**

In the presence of DMSO and SAM or SIN, TthHB27I is transformed from rare 6-bp cutter to a very frequent one, approximately 3-bp. Thus, TthHB27I/SIN(SAM) comprises a new tool in the very low-represented segment of such prototype REases specificities. Moreover, this modified TthHB27I enzyme is uniquely suited for controlled DNA fragmentation, due to partial DNA cleavage, which is an inherent feature of the *Thermus*-family enzymes. Such tool can be used for quasi-random libraries generation as well as for other DNA manipulations, requiring high frequency cleavage and uniform distribution of cuts along DNA.

**Electronic supplementary material:**

The online version of this article (10.1186/s12864-018-4748-0) contains supplementary material, which is available to authorized users.

## Background

Current, strong emphasis in biomedical and molecular biology research is set on whole genomes and metagenomes sequencing, due to the recent substantial reductions in DNA sequencing costs and technological advances. This includes the personalised and precision medicine approach [1] and the Earth BioGenome Project aiming at sequencing genomes of all life forms, among others. Several Next Generation Sequencing (NGS) platforms require employment of DNA fragmentation technologies, appropriate for downstream modification of high molecular weight (HMW) DNA starting material [2], which comes from various sources: genomic libraries, long-range PCR products, cDNA, genomic and metagenomic DNA [3]. From initial sources DNA libraries and/or PCR matrices are prepared. Although rapid development of NGS is observed, factors such as genomic contig assembly and generation of representative libraries often limit speed and accuracy of whole genomes complete sequencing and assembly in continuous contig. Available DNA fragmentation methods include hydrodynamic shearing [4], sonication [5], DNase I fragmentation [6], atomization [7], nebulization [8], point-sink shearing [9]. Unfortunately, all these methods have various drawbacks. These include DNA damage, difficulties in fragmentation control, irreproducibility and non-overlapping DNA segment content in prepared DNA sample and some are difficult to automate. Improvements in these limited DNA scission methods are consequently required. An alternative method for obtaining higher quality DNA fragments involves the use of easily controlled enzymes, which also yield defined and uniform DNA ends, such as partial digestion with REases. For those purposes ideal REase would cleave DNA very frequently to generate overlapping DNA fragments, but also be prone to generate partial digests, not only by employing limiting reaction conditions, such as using low enzyme concentration, short reaction time, inclusion of inhibitors etc., but having natural tendency toward generation of partial digests. In nature, however, very frequent ‘cutters’ are extremely rare. Out of all known site-specific REases prototypes (482 found thus far [10]), the only REases recognising/cleaving DNA sequences shorter than 4 bp are: CviJI/CviJI* [11], SetI [12] and FaiI [10]. This unusual feature make them unique molecular tools for the development of representative DNA libraries. However, these REases efficiently cleave DNA, which makes control of partial digests not simple task. Biotechnology research has led to development of artificial frequent ‘cutter’ such as an enzyme mixture - NEBNext dsDNA Fragmentase (New England Biolabs, Ipswich, MA, USA). In this method dsDNA breaks are produced by the concerted action of two enzymes, with one enzyme randomly nicking dsDNA, and the other recognizing the nicked site and cutting the DNA strand opposite the nick. We have developed an alternative approach, using TspGWI and TaqII REases-MTases, which naturally recognise relatively long 5–6-bp DNA sequences, but when combined with SAM cofactor analogue – SIN, they cleave DNA much more frequently. This can be further enhanced by combination of SIN with DMSO in one reaction, leading to conversion of TspGWI and TaqII to approx. 3-bp frequent ‘cutters’ of different specificities [13, 14]. As SAM/SIN binding motifs (DPACGSG, PPACGSG, DPAVGTG or DPAMGTG) within those enzymes are located far away from REase catalytic motif (atypical D-EXE or PDX_13_EX_1_K) [15], and since both are not involved in DNA recognition, the specificity change must occur through remote distance via allosteric proteins conformation change, which alters DNA contacts within amino acid residues engaged in sequence recognition. This phenomenon can be distinguished as cofactor/analogue-induced ‘star’ activity. The classic ‘star’ activity phenomenon is well known for several decades, back to 70s and early 80s, as relaxation of DNA recognition specificities of REases, caused by altered reaction conditions, such as departing from optimum pH, salt concentration, magnesium ions concentration or replacing them by some other divalent cations, the presence of organic solvents or excessive REase concentration [16–19]. This work is based on the discovery of a new type of ‘star’ activity by our group, which is triggered by binding of the cofactor SAM or its analogues to sub-Type IIG REases [13, 14]. Since the *Thermus*-family enzymes have natural tendency to yield highly variable mixture of partial digests, they are ideal for overlapping DNA fragments generation. In this report we present the third new prototype specificity of this kind: TthHB27I/SIN(SAM), cleaving DNA with a frequency corresponding to approx. 3-bp enzyme as well as novel aspects of SAM and SIN effect on REases-MTases: unlike other enzymes of the family, DMSO is required for induction of cofactor/analogue-induced ‘star’ activity and capability of cofactor SAM to also induce ‘star’ activity. This new, artificial ‘molecular scissor’ is potentially very useful for generating alternative sets of quasi random genomic libraries. Besides a practical application of the developed technology, this report also sheds some light on an enzyme-DNA interaction form the perspective of basic research.

## Methods

### Bacterial strains, plasmids, media and reagents

For DNA purification and cloning of lambda DNA fragments *E. coli* TOP10 {F^−^ *mcr*A Δ(*mrr*-*hsd*RMS-*mcr*BC) φ80*lac*ZΔM15 Δ*lac*X74 *nup*G *rec*A1 *ara*D139 Δ(*ara-leu*)7697 *gal*E15 *gal*K16 *rps*L(Str^R^) *end*A1 λ^−^} (Invitrogen, Carlsbad, CA, USA) was used. Bacteria were grown in LB medium [20] supplemented with ampicillin (100 μg/ml). Components of the media used [20] were from BTL (Lodz, Poland). Agarose was from FMC (Rockland, NY, USA). DNA isolation kit (GeneJet Plasmid Miniprep Kit), DNA markers (GeneRuler 1 kb DNA Ladder, 100 bp Plus DNA Ladder), SmaI REase, FastAP Thermosensitive Alkaline Phosphatase, T4 DNA Polymerase and T4 DNA Ligase were from Thermo Fisher Scientific/Fermentas (Vilnius, USA/Lithuania). The proofreading Marathon DNA Polymerase was from A&A Biotechnology (Gdynia, Poland). Other reagents were from Avantor Performance Materials Poland S.A. (Gliwice, Poland), Sigma-Aldrich (St. Louis, MO, USA), AppliChem Inc. (St. Louis Missouri, MO, USA) or Fluka Chemie GmbH (Buchs, Switzerland). The oligodeoxyribonucleotide (oligo) chemical synthesis and DNA sequencing services were conducted at Genomed S.A. (Warsaw, Poland). Recombinant TthHB27I was purified to homogeneity as we described previously [21].

### Substrate DNA preparation for cleavage assay

PCR fragment (1789 bp) containing two convergent recognition sequences for TthHB27I was amplified from pACYC184 plasmid DNA using a pair of primers: 5’-CATCAGCGCTAGCGGAGTGTA-3′ and 5’-CGAGGGCGTGCAAGATTCC-3′ and Marathon DNA Polymerase. The final volume of the reaction was 100 μl and it contained 35 ng of pACYC184 as a template, 0.5 μM of each primer, 0.4 mM of each dNTPs, 1× Marathon PCR Buffer and 1.5 units of Marathon Polymerase. The cycling profile of reaction included: 97 °C for 4 min, 89 °C for 20 s (addition of the polymerase), 30 cycles of following 3 stages - 94 °C for 30 s, 56 °C for 30 s, 68 °C for 2 min, and as a final step 68 °C for 1.5 min. PCR products were purified by gel electrophoresis, electroelution and ethanol precipitation.

The second PCR product (1850 bp) did not contain any of canonical sequences for TthHB27I (see Additional file 1). To amplify this DNA fragment a pair of primers: 5’-CGCAGAAGGTGTCGGCATATAC-3′ and 5’-GCATCCTGAATGCAGCCATAG-3′, Marathon DNA Polymerase and λ DNA as a template were used. Reactions were performed in final volume of 100 μl and they contained 30 ng of λ DNA, 0.5 μM of each primer, 0.4 mM of each dNTPs, 1× Marathon PCR Buffer and 1.5 units of Marathon Polymerase. The cycling profile included: 95 °C for 2 min, 89 °C for 20 s (addition of the polymerase), 30 cycles of following 3 stages - 95 °C for 30 s, 62 °C for 30 s, 68 °C for 2 min, and as a final step 68 °C for 2 min.

The ability of TthHB27I to cleave methylated DNA was examined using the 1789 bp PCR fragment with the methylated TthHB27I DNA recognition sequences. For this purpose methylation reactions were performed. The reaction mixtures contained 500 ng of DNA, 1× reaction buffer (10 mM Tris-HCl pH 7.0 at 65 °C, 6 mM β-mercaptoethanol (βME), 40 mM NaCl) with 6 mM CaCl_2_, 100 μM SAM, 0.1 mg/ml BSA and 2 U of TthHB27I (4:1 recognition sites to enzyme molecules molar ratio) and were incubated in final volume of 50 μl for 6 h at 65 °C. After that proteinase K was added to the solution and the incubation was carried out for 1 h in 55 °C. In the next step reactions were purified by phenol/chloroform extraction, DNA was ethanol-precipitated and after centrifugation the DNA precipitate was dissolved in water.

### PCR fragment DNA cleavage assay

Two PCR fragments were used to determine the specificity of cofactor/analogue-induced ‘star’ activity of TthHB27I. The first PCR fragment (1789 bp) was used as a substrate DNA to examine the cleavage pattern of TthHB27I digestion in the presence of DMSO without and with addition of SAM or one of its analogues: SIN, S-adenosyl-L-cysteine (SAC), S-adenosyl-homocysteine (SAH) and an analogue of an adenosyl-portion of SAM - ATP. PCR fragment containing two convergent (→←) recognition sequences for TthHB27I (both 5’-CAAACA-3′ and 5’-CAAGCA-3′) was amplified from pACYC184 plasmid DNA (see Additional file 1). The second PCR product did not contain any of canonical sequences for TthHB27I. The substrate was amplified from λ DNA and was 1850 bp long (see Additional file 1). It was used to examine whether TthHB27I cleaves DNA under relaxation conditions, if there are no canonical enzyme recognition sites in DNA molecule. The ability of TthHB27I to cleave methylated DNA was examined using the 1789 bp PCR fragment with the methylated TthHB27I DNA recognition sequences. For this purpose methylation reactions were performed as described above. The obtained methylated substrate DNA was used to examine the cleavage pattern and conditions allowing cofactor/analogue-induced ‘star’ activity of TthHB27I. The reactions contained 500 ng of DNA, 1× REase buffer (10 mM Tris-HCl pH 7.0 at 65 °C, 6 mM βME, 40 mM NaCl, 6 mM MgCl_2_, 0.1 mg/ml BSA, with added 100 μM SAM or its analogue, 2 U (0.76 μg) of TthHB27I and DMSO (*v*/v) in various concentration. Mixtures were incubated in 65 °C for 1 h in final volume of 50 μl.

### λ DNA cleavage assay, shotgun fragments generation and cloning

For recognition sequence and cleavage sites determination λ DNA was used. Cleavage of DNA was carried out in the REase buffer in the volume of 50 μl. The reactions contained 500 ng of DNA, 100 μM SAM or SIN, 2 U of TthHB27I (molar ratio of recognition sites to enzyme molecules was 4:1) and 25% (*v*/v) DMSO. Mixtures were incubated for 4 h in 65 °C, then proteinase K was added and the mixture was further incubated for 1 h in 55 °C. DNA fragments were purified by phenol/chloroform extraction and precipitation with ethanol. In general, a brief purification of *Thermus*-family enzymes-digested DNA prior to electrophoresis substantially improves clarity of digestion patterns seen on a gels, as these are strong DNA binding proteins, used at high concentrations. The DNA precipitates were centrifuged, dissolved in water and treated with T4 DNA polymerase in the presence of dNTPs (0.33 mM each). After purification by proteinase K digestion, phenol/chloroform extraction and ethanol-precipitation, λ DNA fragments with blunt ends were cloned into pUC19. As a vector pUC19 plasmid DNA was used, which enables easy detection of positive clones. Plasmid DNA was digested with SmaI REase and dephosphorylated with the use of FastAP Thermosensitive Alkaline Phosphatase. After dephosphorylation, vector was purified by gel electrophoresis, electroelution, phenol/chloroform extraction and ethanol precipitation. Ligation of λ DNA fragments and vector was performed with T4 DNA ligase. DNA was then phenol/chloroform extracted and ethanol-precipitated.

### Determination of TthHB27I cofactor/analogue-induced ‘star’ recognition and cleavage sites

The DNA obtained by ligation was transformed into *E. coli* TOP10 and plated onto IPTG/X-gal plates. About 200 white colonies were chosen randomly and over 100 clones from each library with the insert size below 1 kb were PCR amplified. Plasmid DNA were isolated and sequenced at the junction of the vector with the insert. Received data were analysed with the use of ABI Chromas 1.45 software (Perkin Elmer Applied Biosystems, Monza, Italy), BLAST [22, 23] and SnapGene [24]. For clones, TthHB27I digestion and PCR products analysis agarose gels were prepared in TBE buffer [20] in concentration depending on the size of DNA used in the reaction. The gels were stained with ethidium bromide or for Fig. 5 with Sybr Gold (Thermo Fisher Scientific/Invitrogen, USA), visualized using a 312 nm UV transilluminator and photographed with a photographic filter.

## Results and discussion

### Detection and optimization TthHB27I cofactor/analogue-induced ‘star’ activity

**Fig. 1:**
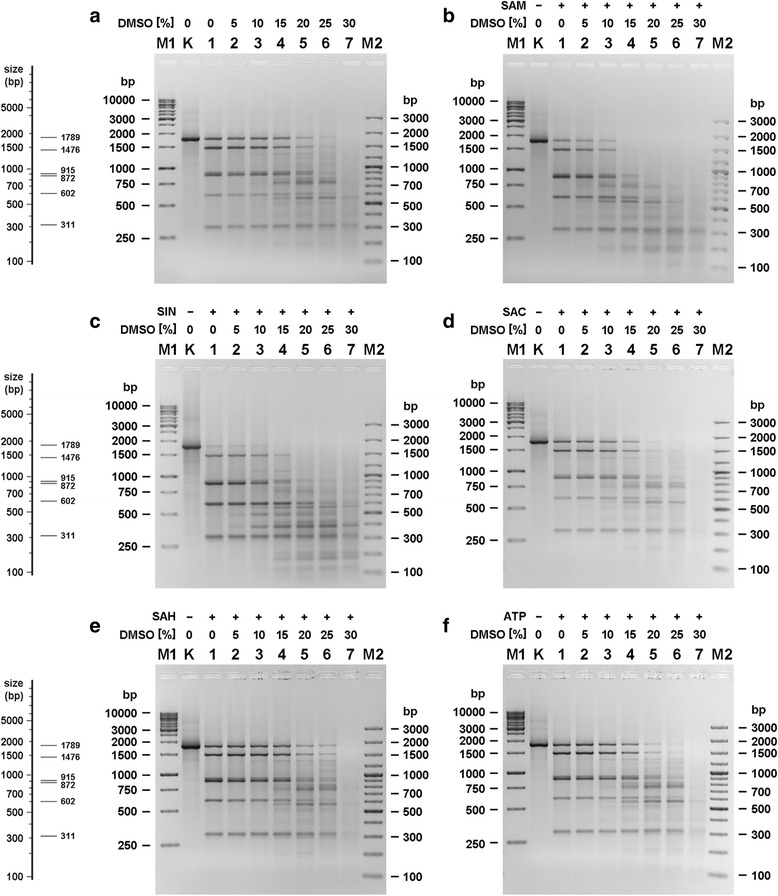
TthHB27I digestion patterns comparison in the presence of DMSO and cofactor SAM or its analogues. 0.5 μg of 1789 bp PCR DNA substrate (see Additional file 1) was incubated with 2 U of TthHB27I for 1 h at 65 °C in REase buffer with increasing amount of DMSO. Reaction mixtures were precipitated and electrophoresed in 1.3% agarose/TBE gels. The pictures on the right side of the figure show the theoretical arrangement of the DNA bands in an agarose gel after digesting the substrate DNA with TthHB27I under standard conditions. **a** Digestions performed only with the addition of DMSO, without any cofactor. Lane M1, GeneRuler 1 kb DNA Ladder; lane M2, 100 bp Plus DNA Ladder; lane K, undigested PCR fragment; lane 1, 0% (*v*/v) DMSO present in the reaction mixture; lane 2, with 5% (v/v) DMSO; lane 3, with 10% (v/v) DMSO; lane 4, with 15% (v/v) DMSO; lane 5, with 20% (v/v) DMSO; lane 6, with 25% (v/v) DMSO; lane 7, with 30% (v/v) DMSO. As in (**a**), but in the presence of 100 μM SAM (**b**); 100 μM SIN (**c**); 100 μM SAC (**d**); 100 μM SAH (**e**); 100 μM ATP (**f**)

We have previously described [25] that TthHB27I from *Thermus thermophilus* HB27 - a member of the *Thermus-*family [26] - recognizes two canonical 6-bp sequences (5’-CAAACA-3′ and 5’-CAAGCA-3′) and cleaves DNA strands at a distance of 11^th^/9^th^ nt downstream of the cognate site. Initially we have shown that SAM and its analogue SIN both have stimulatory effect on TthHB27I REase activity [25]. However, under the conditions studied, both SIN and SAM do not induce the recognition sequence specificity change [25], as opposed to TspGWI [13] and TaqII [14]. To precisely determine TthHB27I cofactor/analogue-induced ‘star’ activity requirements, we devised dedicated DNA substrates: (i) containing two 6-bp cognate sites (1789 bp) and (ii) devoid of cognate recognition sites (1850 bp). The 1789 bp fragment contained both variants of the degenerated, canonical TthHB27I recognition sequences, oriented convergently (see Additional file 1). Under optimal conditions for non- cofactor/analogue-induced ‘star’ activity condition, the expected digestion pattern of TthHB27I on 1789 bp substrate includes a mixture of 6 DNA fragments (311, 602, 872, 915, 1476, 1789 bp), containing complete and partial digestion products, as DNA cleavage with TthHB27I does not go to completion, just like for other *Thermus*-family enzymes (Fig. 1). Figure 1 reveals that the addition of DMSO to the reaction causes specificity relaxation of the enzyme, just as classic ‘star’ activity (Fig. 1a). However, supplementing TthHB27I reactions with both DMSO and SIN or SAM leads to further, intense relaxation of specificity, as a result of cofactor/analogue-induced ‘star’ activity (Fig. 1b, c). With the higher concentrations of DMSO added into DNA scission reactions we observed the increasing number of DNA digestion fragments (Fig. 1a). Thus, TthHB27I is highly prone to DNA cleavage recognition change by addition of DMSO alone, as in regular ‘star’ activity, observed for orthodox REases. We further evaluated potential synergistic effect between DMSO and simultaneously added cofactor SAM or its analogue SIN. Those experiments shown that addition of either SAM or SIN in combination with DMSO results in synergistic effect, observed as increased specificity relaxation (Fig. 1b, c). This effect was not seen when SAC, SAH or ATP where added to the reaction, instead of SAM or SIN (Fig. 1, d, e, f). In the absence of DMSO, SAC/SAH/ATP were also inert in their effect on non-cofactor/analogue-induced ‘star’ activity reactions, e.g. not accelerated TthHB27I activity toward cleavage of cognate sites [25]. The strongest synergistic effect was observed in the DMSO concentration range of 20–30% (*v*/v) (Fig. 1b, c). However, 30% (v/v) DMSO (or higher) in the reaction typically leads to a reduced solubility of the digested DNA fragments. For that reason, for further study we used DMSO at a concentration of 25% (v/v). Even though all REases from *Thermus*-family of homologous REases-MTases [26] are affected by SAM and SIN, there are substantial differences: (*i*) REase activity stimulation without induction of cofactor/analogue-induced ‘star’ activity, (*ii*) slight REase inhibition, (*iii*) cofactor/analogue-induced ‘star’ activity, which either requires or does not require reaction buffer conditions modifications [13–15]. For TthHB27I there are two major novel aspects of cofactor/analogue-induced ‘star’ activity: (*i*) unlike other enzymes of the family, DMSO addition is obligatory for induction of cofactor/analogue-induced ‘star’ activity and (*ii*) capability of SAM to also induce ‘star’ activity, although to lower extent (Fig. 1b; see Additional file 2).

### The effect of presence/absence of cognate recognition sequences in substrate DNA on TthHB27I cofactor/analogue-induced ‘star’ activity

**Fig. 2:**
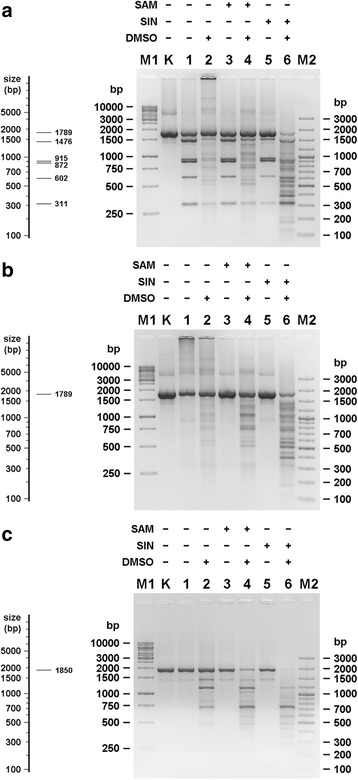
TthHB27I cofactor/analogue-induced ‘star’ activity towards methylated or non-methylated substrate DNA. 0.5 μg of methylated or non-methylated PCR DNA fragment (see Additional file 1) was digested with 2 U of TthHB27I in REase buffer supplemented with 100 μM of the selected effector and 25% (v/v) DMSO at 65 °C. The pictures on the right side of the figure’s panels show the theoretical arrangement of the DNA bands in an agarose gel after digesting the given substrate DNA with TthHB27I under standard conditions. **a** Cleavage pattern of non-methylated 1789 bp PCR fragment DNA. Lane M1, GeneRuler 1 kb DNA Ladder; lane M2, 100 bp Plus DNA Ladder, lane K, untreated DNA; lane 1, cleavage reaction in the absence of effector and DMSO; lane 2, in the presence of DMSO only; lane 3, in the presence of SAM only; lane 4, in the presence of SAM and DMSO; lane 5, in the presence of SIN only; lane 6, in the presence of SIN and DMSO. **b** The same as (**a**), but with previously methylated 1789 bp PCR fragment. **c** The same as (**a**), but with 1850 bp PCR fragment without TthHB27I cognate recognition sequence

To evaluate an effect of the presence of TthHB27I cognate recognition sites on cofactor/analogue-induced ‘star’ activity DNA cleavage we conducted cleavage reactions using two variants of 1789 bp PCR product (see Additional file 1): unmodified (Fig. 2a) and pre-methylated by TthHB27I MTase within the two cognate sites (Fig. 2b). To rule out rather remote possibility, that methylated sites are capable of specific interaction with TthHB27I and stimulation of cofactor/analogue-induced ‘star’ activity, even though they are not cut, we also used the 1850 bp PCR fragment (see Additional file 1), devoid of the canonical sequences (Fig. 2c). One could expect essentially two scenarios: (*i*) the presence of cognate site imposes stimulatory effect and/or is obligatory for DNA cleavage with cofactor/analogue-induced ‘star’ activity mode or (*ii*) relaxed cofactor/analogue-induced ‘star’ activity recognition sequences are cleaved independently of cognate sites. For a DNA library preparation purposes possibility (*ii*) would be preferred, as leading to more randomised, not nested, distribution of cleavages along DNA molecules. As there is no significant difference in cleavage patterns between Fig. 2a, lanes 4, 6 and Fig. 2b, lanes 4, 6 one can draw conclusion, that TthHB27I methylation of cognate sequences has no effect on cleavage, thus most probably cofactor/analogue-induced ‘star’ sites are cut independently of cognate sites. This was further reinforced by the 1850 bp PCR fragment cleavage seen at Fig. 2c, lanes 4, 6. As in Fig. 1, for all substrates variants cases digestion of DNA was observed also in reactions with DMSO added (Fig. 2a, b, c, lanes 2), which further increased with addition of SAM or SIN. Taken together, the results indicate that the lack of functional recognition sequences in digested DNA (absence or methylation) does not affect the cofactor/analogue-induced ‘star’ activity of TthHB27I, as was the case of TspGWI/SIN and TaqII/SIN.

### Determination of TthHB27I recognition sequence and cleavage site under cofactor/analogue-induced ‘star’ activity conditions

**Fig. 3:**
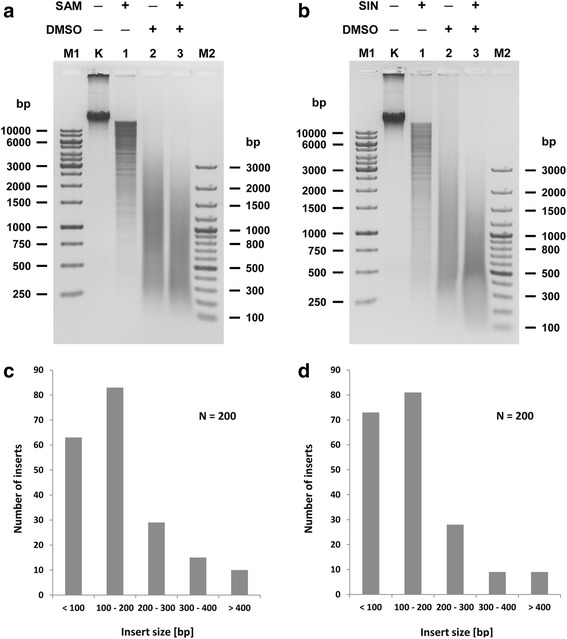
Cleavage of λ DNA under TthHB27I specificity relaxation conditions. **a** Cleavage pattern of λ DNA digested with TthHB27I under various conditions. 0.5 μg of DNA substrate was digested with 2 U of TthHB27I in REase buffer supplemented with 100 μM SAM and/or 25% (v/v) DMSO for 4 h at 65 °C. Lane M1, GeneRuler 1 kb DNA Ladder; lane M2, 100 bp Plus DNA Ladder; lane K, undigested DNA; lane 1, digestion in the presence of SAM; lane 2, digestion in the presence of DMSO; lane 3, digestion in the presence of both SAM and DMSO. **b** The same as (**a**), but SIN was used instead of SAM. **c** Distribution of insert lengths in 200 clones randomly selected from TthHB27I/DMSO/SAM-generated λ DNA library. **d** The same as (**c**), but for TthHB27I/DMSO/SIN-generated λ DNA library

**Fig. 4:**
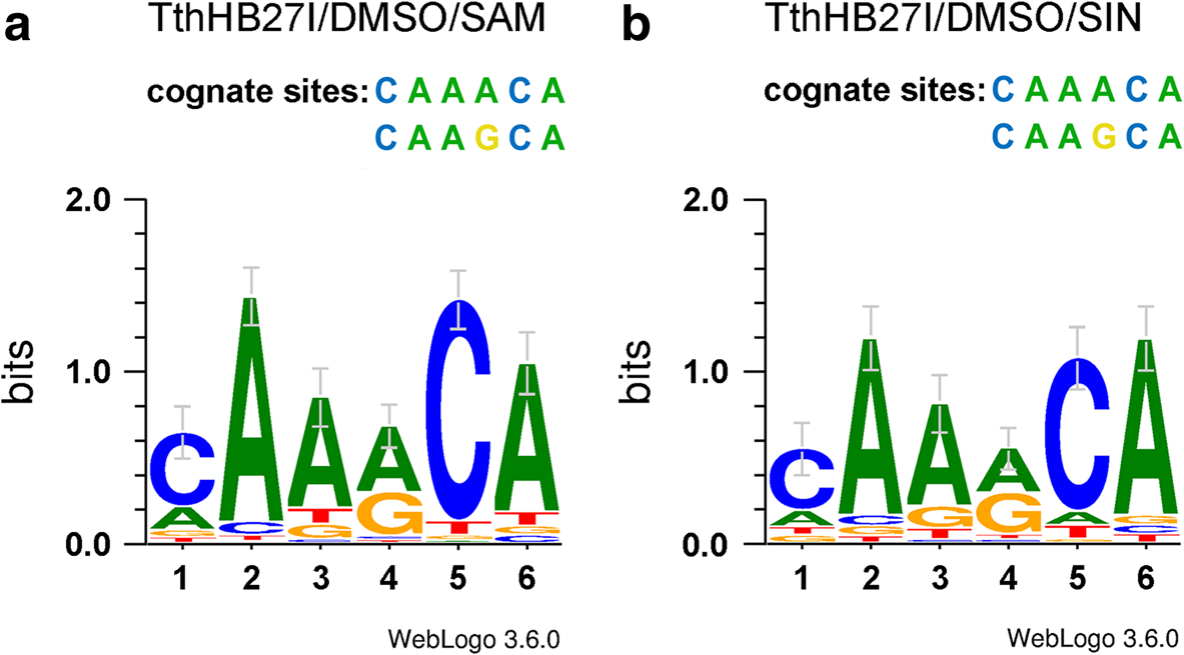
WebLogos of TthHB27I cofactor/analogue-induced ‘star’ activity recognition sequences. The WebLogo graphical sequences were constructed as based on the results of 465 cofactor/analogue-induced ‘star’ recognition sequences shown as tables in Additional files 2 and 3. **a** WebLogo for combined TthHB27I/DMSO/SAM recognition sequences. **b** WebLogo for combined TthHB27I/DMSO/SIN recognition sequences

**Fig. 5:**
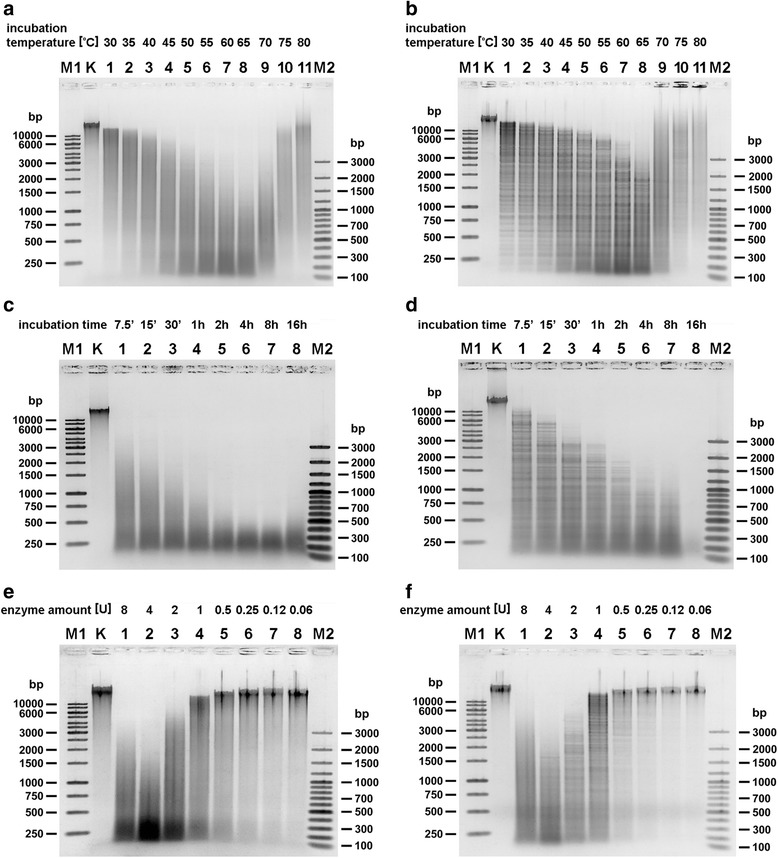
Controlling of partial cleavage of genomic DNAs by TthHB27I under cofactor/analogue-induced ‘star’ activity conditions. *E. coli* and λ genomic DNAs were digested with TthHB27I under cofactor/analogue-induced ‘star’ conditions, while varying: temperature, incubation time and TthHB27I amount. 0.5 μg of DNAs were digested with TthHB27I in REase buffer supplemented with 100 μM SIN and 25% (v/v) DMSO. **a** Cleavage control by reaction temperature. Reactions conducted with *E. coli* DNA digested with 2 U of TthHB27I for 1 h at temperatures from 30 °C to 80 °C. Lane M1, GeneRuler 1 kb DNA Ladder; lane M2, 100 bp Plus DNA Ladder; lane K, undigested DNA; lane 1, reaction at 30 °C; lane 2, 35 °C; lane 3, 40 °C; lane 4, 45 °C; lane 5, 50 °C; lane 6, 55 °C; lane 7, 60 °C; lane 8, 65 °C; lane 9, 70 °C; lane 10, 75 °C; lane 11, 80 °C. **b** The same as (**a**), except that λ DNA was used. **c** Cleavage control by reaction time. *E. coli* DNA was digested at 65 °C at times ranging from 7.5 min to 16 h. Lane M1, GeneRuler 1 kb DNA Ladder; lane M2, 100 bp Plus DNA Ladder; lane K, undigested DNA; lane 1, reaction conducted for 7.5 min; lane 2, 15 min; lane 3, 30 min; lane 4, 1 h; lane 5, 2 h; lane 6, 4 h; lane 7, 8 h; lane 8, 16 h. **d** The same as (**c**), except that λ DNA was used. **e** Cleavage control by the enzyme amount. *E. coli* DNA was digested at 65 °C with TthHB27I. Lane M1, GeneRuler 1 kb DNA Ladder; lane M2, 100 bp Plus DNA Ladder; lane K, undigested DNA; lane 1, DNA digested with 8 U TthHB27I; lane 2, 4 U; lane 3, 2 U; lane 4, 1 U; lane 5, 0.5 U; lane 6, 0.25 U; lane 7, 0.12 U; lane 8, 0.06 U. **f** The same as (**e**), except that λ DNA was used

To examine the relaxed specificity of TthHB27I/DMSO/SAM and TthHB27I/DMSO/SIN, two independent libraries of λ DNA fragments were prepared using the shotgun cloning technique. For this purpose λ DNA was digested with TthHB27I in the presence of 25% (*v*/v) DMSO and SAM (Fig. 3a). The second reaction contained SIN instead of SAM (Fig. 3b). Resulting DNA fragments were cloned into SmaI site in pUC19 vector. Obtained clones were sequenced using the vector’s standard primers to determine sequences around junctions between inserts and vector. 153 clones from TthHB27I/DMSO/SAM library and 139 clones from TthHB27I/DMSO/SIN library were investigated and total of 465 sites variants were analysed (see Additional files 2 and 3). Sequencing has shown that some clones contained recombinant pUC19 with several inserts at a time. 48 and 66 TthHB27I relaxed recognition sites variants were detected, respectively (see Additional files 2 and 3). In both cases, the detected recognition sequence variants had one (app. 50%) or two (app. 50%) nt changed within 6-nt 5’-CAARCA-3′ canonical sequence. Most often, the nt changes were observed at positions 1, 3 and 6 of the 5’-CAARCA-3′ site. Thus, both TthHB27I/DMSO/SAM and TthHB27I/DMSO/SIN interaction with DNA prefers relatively unchanged 2^nd^ bp and 4^th^–5^th^ bp core segment of the cognate site, although it is not an obligatory requirement (see Additional files 2, 3, Fig. 4). Figure 4 presents Web Logo graphic ranking that allows visualization of preferred bases within recognition sites, similar to that presented for ranking of cleavage sites for frequently cleaving, methyl-dependent endonuclease Mcr [27–29]. As evident from Fig. 4, the combined cofactor/analogue-induced ‘star’ recognition ‘site’ is the same for both TthHB27I/DMSO/SAM and TthHB27I/DMSO/SIN. Fig. 3c, d shows the length distribution of the digestion products based on the inserts lengths present in the analyzed clones. The majority of them falls into a category of less than 200 bp, as the experiment described in Fig. 3 was conducted under conditions to obtain maximum possible DNA digestion in order to allow the sequencing both insert-vector junctions. Even under those overdigestion conditions, partial digests still vastly dominate the obtained DNA fragments pool, pointing into the enzyme suitability for easily controlled, partial digestions for genomic library construction purposes. The number of detected cofactor/analogue-induced ‘star’ activity clones reflects the average occurrence of DNA recognition event: (*i*) every 81.9-bp for TthHB27I/DMSO/SAM (4096/48 variants + 2 canonical), if complete cleavage would be taking place, and (*ii*) every 60.2-bp for TthHB27I/DMSO/SIN (4096/66 + 2). Thus, combined, theoretical length of the recognized sequence is: (*i*) 3.2-bp for TthHB27I/DMSO/SAM and (*ii*) 3.0-bp for TthHB27I/DMSO/SIN. This indicates great functional length reduction of the canonical 6-bp cognate site. We sequenced 292 clones, some with multiple inserts, thus total of 465 relaxed sites were analyzed. We cannot be sure that we discovered all the variants, although we believe that we have reached a plateau as no more variants appeared after app. 80% clones sequenced. In general, however, new variants, if any, would not change the major point of this work – developing of a new approx. 3-bp specificity for ultra-frequent DNA fragmentation and randomized genomic libraries construction. If more variants will be discovered in the future, a slightly shorter ‘theoretical’ recognition site would make TthHB27I/DMSO/SIN even more useful. We refer to this DNA recognition/cleavage specificity change as ‘theoretical’ because the value of the 3-bp recognition site is based on the combined occurrence of tens of variants of degenerated 6-bp cognate sites, which occur at different probabilities (see Fig. 4 and Additional files 2, 3). Thus, the ‘3-bp recognition site’ is just a statistical equivalent and there is no fixed 3-bp sequence recognized/cleaved by TthHB27I/DMSO/SIN. For all three developed by us ‘ultra-frequent molecular scissors’, the theoretical length of the cofactor/analogue-induced ‘star’ activity recognition site is approx. 3-bp, regardless, whether unrelaxed cognate sites were 5-bp (TspGWI) [13], 6-bp (TaqII) [14] or degenerated in one position 6-bp (TthHB27I). Such frequent DNA recognition is filling-in the gap between ‘classic’ REases [10], homing endonucleases [30], transcription factors [31] and extremely frequently recognizing enzymes/proteins, such as DNase I [32] or single-stranded binding proteins (SSB) [33, 34]. In all developed by us cases of relaxed *Thermus*-family enzymes, the cleavage distance of 11/9 nt has been retained in cofactor/analogue-induced ‘star’ activity sites, resulting in DNA fragments with 2-nt extended 3′ cohesive ends. For TaqII/SIN we have demonstrated, that after ends blunting, the partial digestion mixture is suitable for eukaryotic genomic library preparation [17]. Bearing in mind similar characteristic of the obtained cleavage products: (*i*) approx. 3-bp theoretical recognition site length, (*ii*) 2-nt/3′ cohesive ends, needed to be blunted and (*iii*) partially digested fragments, it is safe to assume that TthHB27I/DMSO/SIN(SAM) is equally adequate for a library preparation, as TaqII/SIN [14]. Moreover, as opposed to regular ‘star’ activity, the cofactor/analogue-induced ‘star’ activity reaction products are not substantially dominated by cognate DNA digestions products, thus bias toward certain recognition sites is dictated by their occurrence in target DNA. Additionally, partial DNA cleavage, which is an inherent feature of the REases from *Thermus-*family, greatly simplifies determination of an enzyme amount and digestion time, needed to obtain planned fragment length distribution for a given application. We evaluated the conditions of various modes of partial digestion to develop ready-to-use working protocols using two types of genomic substrates as examples: small (48.5 kb) λ DNA and larger (4.8 Mb) *E. coli* DNA. Three parameters were selected for evaluation: reaction temperature, incubation time and the relative TthHB27I/DMSO/SIN REase concentration. As evident in Fig. 5, all three parameters are suitable for partial digestions. However, the control of digestion by temperature seems the most suitable as the distribution of the partial digestion bands is the most ‘smooth’ between 30 °C and 80 °C (Fig. 5a, b). Control by digestion time is also useful and leads to a procedure shortened to just several minutes (Fig. 5c, d). The transition between the desired ranges of digestion fragments distribution can be further ‘smoothed’, as we have evaluated two-fold changes in the incubation time. Control by enzyme dilution seems the most prone to overshooting, especially when using glycerol containing enzyme stocks that are difficult to precisely aliquot (Fig. 5e, f). Thus, the recommended two simple protocols for controlled, partial digestion for library construction purposes are as follows: (*i*) Incubation temperature control: digestion of 0.5 μg of genomic DNA substrate with TthHB27I in 50 μl of “cofactor/analogue-induced ‘star’ REase buffer” (10 mM Tris-HCl pH 7.0 at 65 °C, 6 mM βME, 40 mM NaCl, 6 mM MgCl_2_, 0.1 mg/ml BSA), 100 μM SIN, 25% (*v*/v) DMSO for 1 h with 2 U (0.76 μg) recombinant TthHB27I at 30–65 °C (depending on the desired average fragment length); (*ii*) Incubation time control: digestion of 0.5 μg of genomic DNA substrate with TthHB27I in 50 μl the buffer with 2 U recombinant TthHB27I at 65 °C for 7.5 min (or less) to 1 h (depending on the desired average fragment length). Further digestion processing is conducted by standard protocols, e.g. DNA purification, cohesive ends blunting and cloning. Besides libraries, this new DNA frequent ‘cutter’ can also be applied to other DNA manipulation methods, such as ultrasensitive DNA labelling/amplification, high resolution restriction mapping, RFLP, single-copy genes amplifications, metagenomics, and detection/identification of pathogenic microorganisms without culturing, among others.

## Conclusions

TthHB27I bifunctional REase-MTase, recognising two cognate 6-bp sequences in DNA, was shown to undergo DNA recognition/cleavage specificity change in the presence of cofactor SAM or its analogue SIN, if reactions are supplemented with DMSO. The relaxed, combined recognition site length is approx. 3.2-bp for TthHB27I/DMSO/SAM and approx. 3.0-bp for TthHB27I/DMSO/SIN. Such frequent cutters are very rare. Only three natural REases of such high cleavage frequency are known (CviJI/CviJI*, SetI and FaiI). We have generated artificial specificities: TspGWI/SIN, TaqII/SIN and in this work – TthHB27I/DMSO/SIN(SAM), which comprises a new genomic tool for representative libraries generation, with its usefulness also in other DNA manipulation technologies, requiring fragmentation with high frequency and/or highly randomised cleavage.

## Additional files

Additional file 1: PCR fragment DNA substrates nucleotide sequences. (A) 1789 bp PCR fragment DNA, containing two convergent (→←) TthHB27I canonical sites. Recognition sequence is indicated in bold and underlined. Arrows indicate the cleavage points. Restriction fragments lenght: 311, 602 and 872 bp. (B) 1850 bp PCR fragment DNA without TthHB27I site. (TIF 673 kb)

Additional file 2: TthHB27I specificity change in the presence of SAM and DMSO. (PDF 26 kb)

Additional file 3: TthHB27I specificity change in the presence of SIN and DMSO. (PDF 34 kb)

## Abbreviations

approx: Approximately
bp: Base pair
BSA: Bovine serum albumin
DMSO: Dimethyl sulfoxide
LB: Luria Broth or Lysogenic Broth
min: Minute/minutes
MTase: Methyltransferase
NGS: Next Generation Sequencing
nt: Nucleotide
PCR: Polymerase Chain Reaction
REase: Restriction endonuclease
SAC: S-adenosyl-L-cysteine
SAH: S-adenosyl-L-homocysteine
SAM: S-adenosyl-L-methionine
SIN: Sinefungin
U: Unit(s)
βME: β-mercaptoethanol
