# Supplementary figures and images for: Randomized DNA libraries construction tool: a new 3-bp ‘frequent cutter’ TthHB27I/sinefungin endonuclease with chemically-induced specificity
Source: BMC Genomics. 2018 May 11;19:361. doi: 10.1186/s12864-018-4748-0 (PMC5948728; doi:10.1186/s12864-018-4748-0)

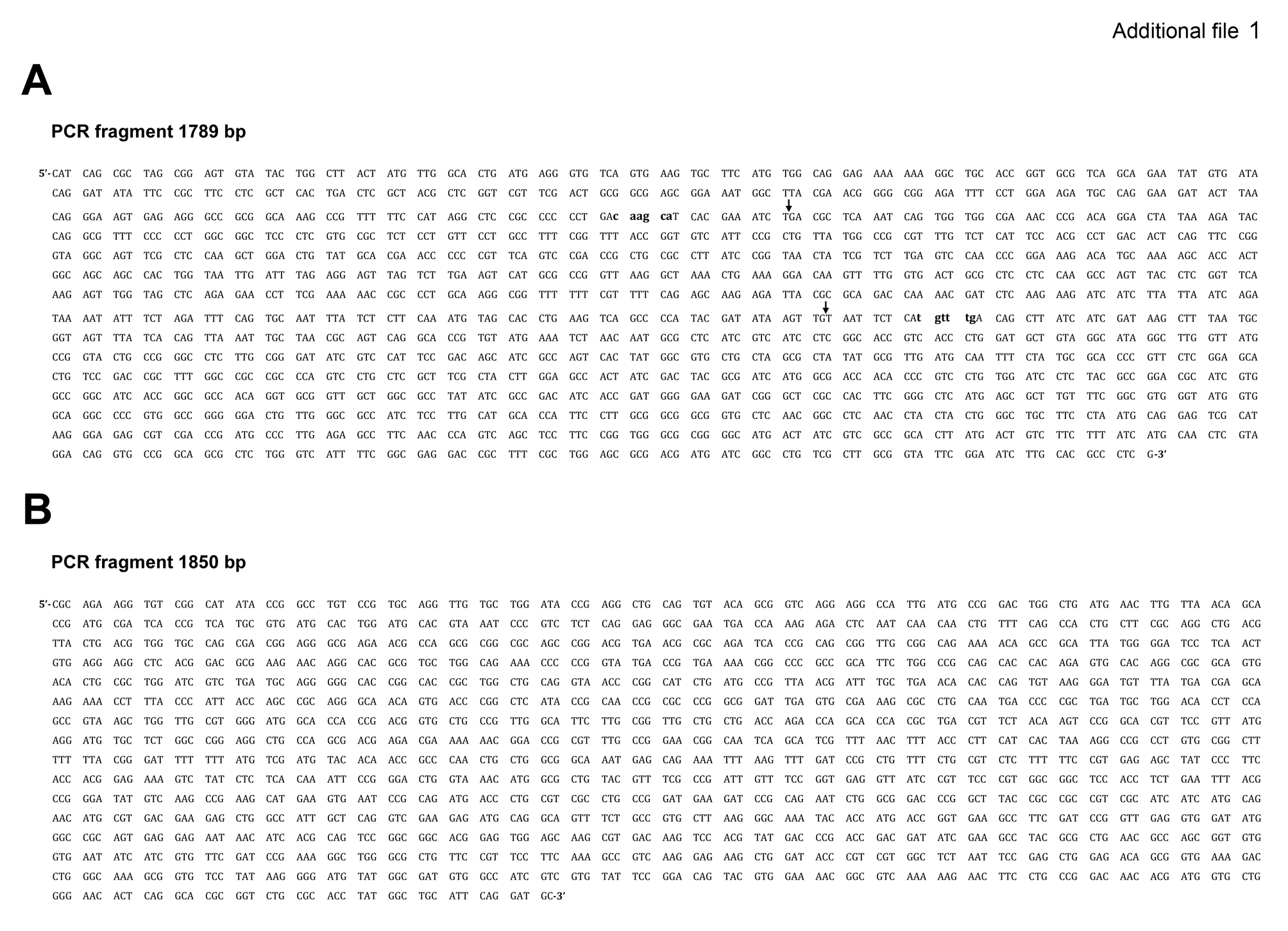

Supplement: Supplementary file 1 — PCR fragment DNA substrates nucleotide sequences. (A) 1789 bp PCR fragment DNA, containing two convergent (→←) TthHB27I canonical sites. Recognition sequence is indicated in bold and underlined. Arrows indicate the cleavage points. Restriction fragments lenght: 311, 602 and 872 bp. (B) 1850 bp PCR fragment DNA without TthHB27I site. (TIF 673 kb) [file 12864_2018_4748_MOESM1_ESM.tif]
