# Supplementary material for: Randomized DNA libraries construction tool: a new 3-bp ‘frequent cutter’ TthHB27I/sinefungin endonuclease with chemically-induced specificity
Source: BMC Genomics. 2018 May 11;19:361. doi: 10.1186/s12864-018-4748-0 (PMC5948728; doi:10.1186/s12864-018-4748-0)
Supplement: Supplementary file 2 — TthHB27I specificity change in the presence of SAM and DMSO. (PDF 26 kb) [file 12864_2018_4748_MOESM2_ESM.pdf]

Table 1 - TthHB27I specificity change in the presence of SAM and DMSO.

variant no									number of sequenced clones	% of total clones	number of changed nucleotides	number of recognition sequences in λ DNA	% of total recognition sequences in λ DNA per variant
TthHB27I													
	5’	C	A	A	A	C	A	3’	22	9.205	-	27	1.870
	5’	C	A	A	G	C	A	3’	7	2.929	-	22	1.524
TthHB27I /SAM/DMSO													
1	5’	**A**	A	A	A	C	A	3’	33	13.808	1	75	5.194
2	5’	**A**	A	A	G	C	A	3’	9	3.766	1	38	2.632
3	5’	**G**	A	A	A	C	A	3’	7	2.929	1	33	2.285
4	5’	**G**	A	A	G	C	A	3’	5	2.092	1	30	2.078
5	5’	**T**	A	A	A	C	A	3’	7	2.929	1	28	1.939
6	5’	**T**	A	A	G	C	A	3’	2	0.837	1	30	2.078
7	5’	C	**C**	A	A	C	A	3’	1	0.418	1	20	1.385
8	5’	C	**C**	A	G	C	A	3’	10	4.184	1	72	4.986
9	5’	C	**G**	A	G	C	A	3’	1	0.418	1	15	1.039
10	5’	C	A	**C**	A	C	A	3’	2	0.837	1	18	1.247
11	5’	C	A	**G**	A	C	A	3’	7	2.929	1	32	2.216
12	5’	C	A	**G**	G	C	A	3’	16	6.695	1	41	2.839
13	5’	C	A	**T**	A	C	A	3’	7	2.929	1	33	2.285
14	5’	C	A	**T**	G	C	A	3’	11	4.603	1	28	1.939
15	5’	C	A	A	**C**	C	A	3’	7	2.929	1	30	2.078
16	5’	C	A	A	**T**	C	A	3’	6	2.510	1	26	1.801
17	5’	C	A	A	A	**A**	A	3’	1	0.418	1	57	3.947
18	5’	C	A	A	A	**G**	A	3’	1	0.418	1	24	1.662
19	5’	C	A	A	G	**G**	A	3’	4	1.674	1	13	0.900
20	5’	C	A	A	A	**T**	A	3’	8	3.347	1	29	2.008
21	5’	C	A	A	G	**T**	A	3’	3	1.255	1	5	0.346
22	5’	C	A	A	A	C	**C**	3’	3	1.255	1	20	1.385
23	5’	C	A	A	G	C	**C**	3’	5	2.092	1	20	1.385
24	5’	C	A	A	A	C	**G**	3’	9	3.766	1	22	1.524
25	5’	C	A	A	G	C	**G**	3’	1	0.418	1	18	1.247
26	5’	C	A	A	A	C	**T**	3’	12	5.021	1	24	1.662
27	5’	C	A	A	G	C	**T**	3’	5	2.092	1	21	1.454
28	5’	**A**	**T**	A	A	C	A	3’	2	0.837	2	32	2.216
29	5’	**A**	A	**T**	A	C	A	3’	2	0.837	2	30	2.078
30	5’	**A**	A	**T**	G	C	A	3’	4	1.674	2	36	2.493
31	5’	**G**	**C**	A	G	C	A	3’	1	0.418	2	75	5.194
32	5’	**G**	**T**	A	A	C	A	3’	1	0.418	2	19	1.316
33	5’	**G**	**T**	A	G	C	A	3’	1	0.418	2	13	0.900
34	5’	**G**	A	A	A	C	**C**	3’	1	0.418	2	34	2.355
35	5’	**T**	**T**	A	A	C	A	3’	1	0.418	2	26	1.801
36	5’	**T**	A	**C**	G	C	A	3’	1	0.418	2	16	1.108

37	5’	**T**	A	**G**	G	C	A	3’	1	0.418	2	9	0.623
38	5’	**T**	A	A	A	C	**T**	3’	1	0.418	2	12	0.831
39	5’	C	**C**	A	A	**T**	A	3’	1	0.418	2	22	1.524
40	5’	C	**T**	**C**	A	C	A	3’	1	0.418	2	18	1.247
41	5’	C	**G**	**T**	A	C	A	3’	1	0.418	2	12	0.831
42	5’	C	A	**C**	**T**	C	A	3’	1	0.418	2	20	1.385
43	5’	C	A	**C**	G	C	**C**	3’	2	0.837	2	34	2.355
44	5’	C	A	**G**	**C**	C	A	3’	1	0.418	2	67	4.640
45	5’	C	A	**T**	A	**A**	A	3’	1	0.418	2	46	3.186
46	5’	C	A	A	A	**A**	**G**	3’	1	0.418	2	26	1.801
47	5’	C	A	A	A	**T**	**G**	3’	1	0.418	2	26	1.801
48	5’	C	A	A	A	**T**	**T**	3’	1	0.418	2	20	1.385

Determination of relaxed recognition sites was conducted by shotgun cloning and sequencing of TthHB27I restriction fragments obtained in the reaction containing SAM and DMSO. TthHB27I – canonical recognition sequences. TthHB27I/SAM/DMSO – variants of relaxed recognition sequences induced by SAM and DMSO. Nucleotides different than those in the canonical sequences are indicated bold and colored.
