# Supplementary material for: Randomized DNA libraries construction tool: a new 3-bp ‘frequent cutter’ TthHB27I/sinefungin endonuclease with chemically-induced specificity
Source: BMC Genomics. 2018 May 11;19:361. doi: 10.1186/s12864-018-4748-0 (PMC5948728; doi:10.1186/s12864-018-4748-0)
Supplement: Supplementary file 3 — TthHB27I specificity change in the presence of SIN and DMSO. (PDF 34 kb) [file 12864_2018_4748_MOESM3_ESM.pdf]

Table 2 -TthHB27I specificity change in the presence of SIN and DMSO.

variant no									numer of sequenced clones	% of total clones	number of changed nucleotides	number of recognition sequences in λ DNA	% of total recognition sequences in λ DNA per variant
**TthHB27I**													
	5'	C	A	A	A	C	A	3'	8	3.540	-	27	1.228
	5'	C	A	A	G	C	A	3'	8	3.540	-	22	1.001
**TthHB27I /SIN/DMSO**													
1	5'	**A**	A	A	A	C	A	3'	19	8.407	1	75	3.412
2	5'	**A**	A	A	G	C	A	3'	10	4.425	1	38	1.729
3	5'	**G**	A	A	A	C	A	3'	6	2.655	1	33	1.501
4	5'	**G**	A	A	G	C	A	3'	5	2.212	1	30	1.365
5	5'	**T**	A	A	A	C	A	3'	3	1.327	1	28	1.274
6	5'	**T**	A	A	G	C	A	3'	7	3.097	1	30	1.365
7	5'	C	**C**	A	A	C	A	3'	1	0.442	1	20	0.910
8	5'	C	**C**	A	G	C	A	3'	5	2.212	1	72	3.276
9	5'	C	**G**	A	A	C	A	3'	2	0.885	1	22	1.001
10	5'	C	**T**	A	A	C	A	3'	3	1.327	1	9	0.409
11	5'	C	A	**C**	A	C	A	3'	1	0.442	1	18	0.819
12	5'	C	A	**C**	G	C	A	3'	1	0.442	1	37	1.683
13	5'	C	A	**G**	A	C	A	3'	13	5.752	1	32	1.456
14	5'	C	A	**G**	G	C	A	3'	14	6.195	1	41	1.865
15	5'	C	A	**T**	A	C	A	3'	7	3.097	1	33	1.501
16	5'	C	A	**T**	G	C	A	3'	6	2.655	1	28	1.274
17	5'	C	A	A	**C**	C	A	3'	6	2.655	1	30	1.365
18	5'	C	A	A	**T**	C	A	3'	9	3.982	1	26	1.183
19	5'	C	A	A	A	**A**	A	3'	4	1.770	1	57	2.593
20	5'	C	A	A	G	**A**	A	3'	4	1.770	1	19	0.864
21	5'	C	A	A	A	**G**	A	3'	3	1.327	1	24	1.092
22	5'	C	A	A	A	**T**	A	3'	7	3.097	1	29	1.319
23	5'	C	A	A	G	**T**	A	3'	5	2.212	1	5	0.227
24	5'	C	A	A	A	C	**C**	3'	2	0.885	1	20	0.910
25	5'	C	A	A	G	C	**C**	3'	2	0.885	1	20	0.910
26	5'	C	A	A	A	C	**G**	3'	8	3.540	1	22	1.001
27	5'	C	A	A	G	C	**G**	3'	2	0.885	1	18	0.819
28	5'	C	A	A	A	C	**T**	3'	4	1.770	1	24	1.092
29	5'	C	A	A	G	C	**T**	3'	1	0.442	1	21	0.955
30	5'	**A**	**C**	A	G	C	A	3'	2	0.885	2	37	1.683
31	5'	**A**	**T**	A	A	C	A	3'	1	0.442	2	32	1.456
32	5'	**A**	A	**C**	G	C	A	3'	1	0.442	2	19	0.864
33	5'	**A**	A	A	G	**A**	A	3'	2	0.885	2	45	2.047
34	5'	**A**	A	A	G	C	**C**	3'	1	0.442	2	37	1.683
35	5'	**A**	A	A	A	C	**G**	3'	1	0.442	2	51	2.320
36	5'	**A**	A	A	G	C	**T**	3'	2	0.885	2	28	1.274
37	5'	**G**	**C**	A	G	C	A	3'	1	0.442	2	75	3.412

38	5’	**G**	**G**	A	G	C	A	3’	1	0.442	2	19	0.864
39	5’	**G**	**G**	A	A	C	A	3’	1	0.442	2	30	1.365
40	5’	**G**	**T**	A	G	C	A	3’	1	0.442	2	13	0.591
41	5’	**G**	A	**G**	G	C	A	3’	1	0.442	2	21	0.955
42	5’	**G**	A	A	**T**	C	A	3’	1	0.442	2	33	1.501
43	5’	**G**	A	A	A	**A**	A	3’	1	0.442	2	70	3.185
44	5’	**G**	A	A	A	C	**C**	3’	1	0.442	2	34	1.547
45	5’	**T**	**C**	A	A	C	A	3’	1	0.442	2	33	1.501
46	5’	**T**	**C**	A	G	C	A	3’	2	0.885	2	61	2.775
47	5’	**T**	**G**	A	A	C	A	3’	1	0.442	2	38	1.729
48	5’	**T**	**G**	A	G	C	A	3’	3	1.327	2	54	2.457
49	5’	**T**	**T**	A	G	C	A	3’	1	0.442	2	12	0.546
50	5’	**T**	A	**T**	A	C	A	3’	1	0.442	2	13	0.591
51	5’	**T**	A	**T**	G	C	A	3’	1	0.442	2	35	1.592
52	5’	C	**G**	**T**	G	C	A	3’	1	0.442	2	22	1.001
53	5’	C	**G**	A	G	**A**	A	3’	2	0.885	2	22	1.001
54	5’	C	**T**	A	A	C	**T**	3’	2	0.885	2	11	0.500
55	5’	C	A	**C**	**T**	C	A	3’	1	0.442	2	20	0.910
56	5’	C	A	**C**	G	C	**C**	3’	1	0.442	2	34	1.547
57	5’	C	A	**C**	G	C	**T**	3’	1	0.442	2	40	1.820
58	5’	C	A	**G**	**C**	C	A	3’	3	1.327	2	67	3.048
59	5’	C	A	**G**	G	**G**	A	3’	1	0.442	2	24	1.092
60	5’	C	A	**G**	A	**T**	A	3’	1	0.442	2	44	2.002
61	5’	C	A	**G**	G	**T**	A	3’	3	1.327	2	28	1.274
62	5’	C	A	**T**	A	**A**	A	3’	1	0.442	2	46	2.093
63	5’	C	A	**T**	G	**A**	A	3’	1	0.442	2	32	1.456
64	5’	C	A	A	**T**	**G**	A	3’	1	0.442	2	26	1.183
65	5’	C	A	A	A	**A**	**C**	3’	2	0.885	2	45	2.047
66	5’	C	A	A	A	**T**	**C**	3’	1	0.442	2	37	1.683

Determination of relaxed recognition sites was conducted by shotgun cloning and sequencing of TthHB27I restriction fragments obtained in the reaction containing SIN and DMSO. TthHB27I – canonical recognition sequences. TthHB27I/SIN/DMSO – variants of relaxed recognition sequences induced by SIN and DMSO. Nucleotides different than those in the canonical sequences are indicated bold and colored.
